# Parental phone use during mealtimes with toddlers and the associations with feeding practices and shared family meals: a cross-sectional study

**DOI:** 10.1186/s12889-021-10757-1

**Published:** 2021-04-20

**Authors:** Frøydis N. Vik, Erik Grasaas, Maaike E. M. Polspoel, Margrethe Røed, Elisabet R. Hillesund, Nina C. Øverby

**Affiliations:** 1 Department of Nutrition and Public Health, University of Agder, Postboks 422, 4604 Kristiansand, Norway; 2 Department of Healthcare and Landscape Architecture, Erasmus Brussels University of Applied Sciences and Arts, Brussels, Belgium

**Keywords:** Parental phone use, Toddlers, Family meals, Meals, Feeding practices, Norway

## Abstract

**Background:**

Positive parental feeding practices and a higher frequency of family meals are related to healthier child dietary habits. Parents play an essential role when it comes to the development of their child’s eating habits. However, parents are increasingly distracted by their mobile phone during mealtimes. The aim of this study was to describe the feeding practices and daily shared family meals among parents who use and do not use a mobile phone during mealtimes, and further to explore the associations between the use of a mobile phone during mealtimes and feeding practices and daily shared family meals, respectively.

**Methods:**

Cross-sectional data from the Food4toddler study were used to explore the association between mobile use during meals and parental feeding practices including family meals. In 2017/2018 parents of toddlers were recruited through social media to participate in the study. In total 298 out of 404 who volunteered to participate, filled in a baseline questionnaire, including questions from the comprehensive feeding practices questionnaire (CFPQ), questions of frequency of family meals and use of mobile phone during meals.

**Results:**

Herein, 4 out of 10 parents reported various levels of phone use (meal distraction) during mealtimes. Parental phone use was associated with lower use of positive parental feeding practices like modelling (B = − 1.05 (95% CI -1.69; − 0.41)) and family food environment (B = − 0.77 (95% CI -1.51; − 0.03)), and more use of negative parental feeding practices like emotional regulation (B = 0.73 (95% CI 0.32; 1.14)) and the use of pressure to eat (B = 1.22 (95% CI 0.41; 2.03)). Furthermore, parental phone use was associated with a lower frequency of daily family breakfast (OR = 0.50 (95% CI 0.31; 0.82)) and dinner (OR = 0.57 (95% CI 0.35; 0.93)).

**Conclusions:**

Mobile phone use is common among parents during mealtimes, and findings indicate that parental phone use is associated with less healthy feeding practices and shared family meals. These findings highlight the importance of making parents aware of potential impacts of meal distractions.

**Trial registration:**

ISRCTN92980420. Registered 13 September 2017. Retrospectively registered.

**Supplementary Information:**

The online version contains supplementary material available at 10.1186/s12889-021-10757-1.

## Introduction

The importance of promoting healthy eating habits early in life cannot be stressed enough. The recent report from the WHO-UNICEF-Lancet commission: *A future for the world’s children*? states that investing in young children’s health, education, and development are fundamental for the individual’s lifelong health and development, and even for their future children’s health [1]. This report also highlights the importance of good health and nutrition in the prenatal period and early years in order to lay the foundation for a healthy life course [1]. An unhealthy diet early in life tends to endure into adulthood [2] and increases the risk of non-communicable diseases (NCD’s) [3] and childhood obesity [4]. Therefore, developing healthy eating habits as a child is essential for maintaining good health throughout life [5]. There are also large societal economic benefits to be made if the population follow dietary guidelines [6].

Different determinants influence the forming of the child’s eating habits. Examples of determinants are family environment, parental food habits, parenting feeding practices, food preferences of the child, family meals, parental educational status and media influences (9.) Parents play a crucial role in establishing healthy eating habits and more generally promoting child development. Parenting style is a behavioral construct that deals with the emotional aspects between how parents and children interact. Two dimensions [7] characterize parenting styles; responsiveness, i.e., parents pay attention to their child in a warm, sensitive, supportive, and respectful way and demandingness, i.e., how much control parents exercise including appropriate expectations for the child’s maturity of feelings and behaviors. There are four various parenting styles that include these dimensions [7]. Authoritative parenting is most aligned with positive parenting because it involves high responsiveness to the child and high level of demandingness and rules. Feeding styles are subcategories of parenting style, related to mealtimes. An authoritative feeding style has in several studies been identified as giving the best potential of foster functional and supportive mealtimes and the right balance of guidance and control that will nurture desirable child eating outcomes [7, 8]. There are three other styles that are less supportive of positive parenting, i.e., authoritarian, indulgent, and uninvolved but they will not be discussed further.

The feeding practices the parents use are also important in developing eating habits for their children and they can promote or restrain a healthy diet [9]. Parental feeding practices are defined by Shloim et al. [7] as specific goal-directed behaviors that parents use to directly influence the children’s eating. In a systematic review and meta-analysis by Yee et al. in 2017, parental feeding practices from 88 empirical studies (of which 66 were cross-sectional, 14 longitudinal and 8 experimental) were reviewed to determine the influence of parents on child food consumption [10]. The parental feeding practices found to be most strongly associated with child food consumption were availability of healthy/unhealthy food at home and parental modeling (e.g., parents’ own food consumption behavior) [10]. Other feeding practices investigated in this systematic review and meta-analysis were not conclusive and they were context-specific: active guidance/education, restrictive guidance/rule-making, accessibility, pressure to eat, rewarding food consumption, using food as reward and finally rewarding with verbal praise which was most evident for children 6 years and younger [10]. Parental feeding practices can be positive or negative in forming eating habits among small children. An example of a negative feeding practice is if parents pressure their child to eat a particular food item (e.g., broccoli), which may lead to disliking of the actual food item (broccoli). An example of a positive feeding practice is involving the child in food preparation (e.g., scrambled eggs) which may increase the willingness to try new food [11]. Parenting styles, feeding styles and feeding practices are constructs to simplify research on parent-child-interactions and food or health outcomes [7]. Responsive feeding, a practice identified by the parent or caregiver responding to the child’s cues in a manner that is emotionally supportive, contingent, and developmentally appropriate - acknowledging the child’s behavior or request, but not necessarily complying with it, is related to healthy eating outcomes [12]. Non-responsive behavior, such as feeding child when not hungry, may lead to impairment of the child’s response to hunger and satiation and is related to overweight [12].

According to Internet Growth Statistics [13], the usage of digital screens, e.g. computers, mobile phones and tablets have increased largely worldwide over the last decades. A majority of people across all socio-economic groups own mobile phones, and it is increasingly considered a “must have” object for almost everyone; richer and poorer. With increasing screen use among the population, our behaviours change. It is common to see people walking outside using their phone, people at restaurants doing the same, even when they are together with other people.

A recent study found that distractions take up a large proportion (almost half) of the average family mealtime [14]. Distractions at mealtimes (e.g. screens) have been shown to be associated with greater intake of unhealthy food [15]. When mealtimes are characterized by disruptions, lack of attention to each other’s moods and eating behaviors, and being away from the table, there is increased risk for poor dietary habits and a stressful eating environment [16, 17]. During mealtimes with no distractions (e.g., no television (TV), phones, toys), preschoolers have been observed engaging in less fussy eating behaviors, whereas mealtimes with a distraction were characterized by more negative child behavior [18]. There is limited research on implications of distractions on parent feeding practices. We hypothesize that since positive feeding practices include responsiveness and paying attention to child cues, spending time on phones, will lead to less healthy feeding practices.

Mealtimes serve as a setting for socialization and forming of food habits and eating behaviours for the child together with their family [19]. Family meals are defined in different ways, depending on the number of family members who are attending (e.g., are one or both parents present?) or if the setting in which the meal takes place is included (e.g., is the meal at the table or in front of the TV?). Verhage et al. uses the definition: The family meal can be seen as a social moment of the day during which food is eaten together with at least one family member [20]. Frequency of family meals have been linked to reduced risk of childhood obesity possibly due to family meals being heathy and varied resulting in better nutritional health [9, 21] and that family meals may be more supervised resulting in healthier eating [22]. A meta-analytic review of 17 studies including more than 180,000 children and adolescents indicated that regularly sharing meals as a family reduced the odds for child overweight by 12% and increased the odds for eating healthy foods by 24% [21]. Parental education level has been shown to be associated with family meals; children of parents with higher educational level were more likely eat regular family breakfast [23]. Family meals may also be an indicator of a health-promoting lifestyle.

Given the importance of positive parent modeling on children’s healthy food habits [10], i.e., family mealtimes are opportunities to provide structure, monitor what children eat, connect emotionally, share enjoyable times and model healthy eating, parental use of a mobile phone during mealtimes may be of concern. There is limited research on implications of distractions on parent feeding practices. Frequency of shared family meals was selected as a relevant outcome due to previously described associations between early family meal participation and nutritional outcomes [20]. We therefore also hypothesize that parental use of mobile phone during mealtimes will be associated with fewer daily shared family meals. Thus, the objective of this paper was to describe the parental feeding practice and frequency of daily shared family meals among parents who use and do not use a mobile phone during mealtimes; and further to explore the association between the use of a mobile phone during mealtimes, feeding practice and daily shared family meals.

## Methods

### Study design

Food4toddlers is originally a randomized controlled trial evaluating the effect of a digital dietary intervention targeting parents of 1-year olds [24]. The current paper reports on cross-sectional baseline data from this study.

### Sample and procedure

In 2017 we recruited parents of toddlers. They were recruited through social media posts on Facebook and the procedure has been published elsewhere [25]. In total, 404 participants volunteered to participate, however of these, only 298 participants filled in the baseline questionnaire. Data from these 298 participants were included in the present study. The study was approved by the Norwegian Centre for Research Data, and by the Faculty ethics committee and has been conducted in line with the Helsinki Declaration of 1985, revised 2008.

### Instruments

The baseline questionnaire (Additional file 1) included questions of parental and child characteristics like age and parental educational status. Further, there were questions regarding use of mobile devices, food frequency questions of child diet and parental diet, frequency of family meals, feeding practices and other intervention specific questions. In the current study the variables use of mobile device during mealtimes (independent variables) and feeding practices and shared family meal frequency (dependent variables) are presented. In addition to parent and child characteristics.

The use of mobile phone during mealtimes, was reported like this: *To what extent do you agree with the following statement: I often check my phone during meals*. The participants were given the following response alternatives: Disagree, slightly disagree, neither agree or disagree, slightly agree and agree. We dichotomized this variable with the intention to differentiate between those who do not use mobile phones during meals at all and those who do. The participants who filled in “disagree” were defined as the “No phone use during meal” group, while the participants who filled in “slightly disagree, neither agree or disagree, slightly agree and agree” were defined as the “phone use during meal” group.

To assess feeding practices, we used the well-known and validated, Comprehensive Feeding Practices Questionnaire (CFPQ) [26]. In a review of 33 individual feeding-related instruments Heller and Mobley [27] considered the CFPQ as one out of three relevant questionnaires that had passed rigorous validation and reliability testing and also one of few suited for children < 2 year of age. The CFPQ questionnaire covers 12 dimensions of parental feeding practices with a total of 44 items assumed to cover these dimensions. Because the participants of this study were parents of toddlers (1-year-olds) the questionnaire was slightly modified to fit this age group. Five items were considered irrelevant to parents of toddlers and were therefore removed: 1) I involve my child in planning family meals; 2) I encourage my child to participate in grocery shopping (Both belonging to the dimension *Involvement*); 3) I encourage my child to eat less so he/she won’t get fat; 4) I often put my child on a diet to control his/her weight (Both belonging to the dimension *Restriction for weight control*); And 5) I discuss with my child the nutritional value of foods (belonging to the dimension *Teaching about nutrition*)). This led us to not present the dimensions *Involvement* and *Teaching about nutrition* in this paper. We therefore assessed the following 10 dimensions of child feeding practices: Child control (The child is allowed to control his/her own eating behaviours, 5 items), Emotion regulation (The parents use food to regulate the child’s emotional states, 3 items:), Balance and variety (Parents encourage well-balanced food intake, 4 items), Environment (Availability of healthy food at home, 4 items), Food as reward (Use of food to reward child behavior, 3 items), Modeling (Parents demonstrate healthy eating behavior, 4 items), Monitoring (Parents keep track of child’s intake of less healthy food, 4 items), Pressure (Used by parents to make child eat more, 4 items), Restriction for health (4 items) and restriction for weight control (Parents control the child’s food intake with the purpose of limiting less healthy foods and sweets and to decrease or maintain the child’s weight, 6 items). Further elaboration of these 10 items can be viewed elsewhere [25]. In line with Musher-Eizenman and Holub the questions had 5-point response scales, either: “never, rarely, sometimes, mostly, and always” or “disagree, slightly disagree, neutral, slightly agree, and agree”. Items were added, and two of the items were reversed, all coding’s were done according to Musher-Eizenman and Holub [26]. The reliability of the 10 feeding practices dimensions was evaluated using Cronbach alpha. All scores except *Child control* and *Food as a reward* (α = 0.3) showed acceptable reliability (α = 0.5–0.8) (detailed data not shown). Due to low internal consistency in *Child control* and *Food as a reward,* we decided to not present these dimensions. The CFPQ items were previously translated from English into Norwegian in another project and a random sample of 10 items were back translated into English [28]. The quality of the translation was considered very good and this translation was therefore used in this study.

To assess the frequency of shared family meals, following questions were posed: How often does your child have the following meals together with their family? Herein, meals with family included parents that were married, living in cohabitant families, single or separated / divorced. The participants could choose from the following response alternatives: Never, 1, 2, 3, 4, 5, 6 and 7 times per week for breakfast, lunch, and dinner, respectively. Later, the weekly food frequency was divided into having every specific family meal, every day or not.

Parental age was assessed in years, child age in months. Participants reported their own and the other parent’s highest level of education (primary school or less, primary schools plus 1 year of further education, high school, vocational school, upper secondary school or less, college/university (≤ 4 years), college/university (> 4 years), other, don’t know). These responses were dichotomized into having no university/college education or at least one parent having university/college education and used as a proxy for socio-economic status. Ethnicity of the parents was assessed as native if they were born in Norway or non-native if they were born elsewhere.

### Statistical analysis

The statistical analyses were conducted using IBM SPSS Statistics for Windows, Version 25.0 (IBM Corp., Armonk, NY). Demographic data were described using descriptive measures. Continuous variables were described with mean and standard deviation, and categorical variables with frequencies and percentages. For the dependent study variable feeding practice, all sub-scales had skewness values of ±1.3 and kurtosis values of ±1.6, which indicated that these variables are approximately normally distributed. Thus, linear regression analyses were conducted between the dependent variables (feeding practices) and the independent dichotomized variable (phone use / no phone use), and then controlled for parental educational status, ethnicity and age using multiple regression analysis. Binary logistic regression analyses were conducted to estimate the relationships between the dependent dichotomized variable shared family meals and the independent dichotomized variable (phone use / no phone use) controlling for parental educational status, ethnicity, and age. Due to the homogeneity of the sample in terms of gender (98.7% were mothers), adjusting for this variable was not considered relevant. *P*-values< 0.05 were considered statistically significant and all tests were two-sided.

## Results

### Participants

The total sample included 298 parents with a mean age of 32.3 (SD ± 4.2) years and their children’s age were 10.9 months (SD ± 0.1). Table 1 presents the descriptive characteristics of the total sample. A great majority of the sample were mothers (98.7%) and consisted of parents who were either married (50.7%), lived in cohabitant families (48.3%) or who were single (0.7%) or divorced / separated (0.3%) at the time of the study. The participants reported primarily a high educational level, herein 53.7% reported more than 4 years of University level. Further, about 4 out of 10 parents reported using a mobile phone during mealtimes with their children. Most parents were born in Norway, i.e., native.

**Table 1:** Descriptive statistics of the sample (*n* = 298)

Characteristics	Values	N (%)
Relationship to child, *N (%)*	Mother	294 (98.7)
	Father	4 (1.3)
Parental education status, *N (%)*	Lower secondary school or less	3 (1.0)
	Upper secondary school	23 (7.7)
	College/University (≤ 4 years)	101 (33.9)
	College/University (> 4 years)	160 (53.7)
	Other	11 (3.7)
Marital status, *N (%)*	Married	151 (50.7)
	Cohabitant	144 (48.3)
	Single	2 (0.7)
	Divorced/separated	1 (0.3)
Ethnicity, *N (%)*	Native	257 (86.2)
	Non-native	41 (13.8)
Child’s gender, *N (%)*	Female	134 (45)
	Male	164 (55)

### Descriptive data of the study variables: phone use, feeding practices and shared family meals

A total of 181 of the participants (60.7%) reported no use of mobile phone during mealtimes, while the remaining 117 participants (39.3%) reported various levels of phone use during mealtimes. The largest difference in mean for feeding practice was shown for the subscale *pressure*, where the group using a phone during mealtimes reported a mean score of 7.1 (SD ± 3.2) compared to 5.8 (SD ± 3.6) in the group with no phone use (Table 2). Higher frequency of all types of shared family meals were reported by the group with no phone use compared to the group with phone use during mealtimes (Table 3). The highest frequency of shared family meals was dinner for both groups (71.3 and 57.3%, respectively) (Table 3).

**Table 2:** Associations between phone use during mealtimes and feeding practices adjusted for parental educational status, ethnicity, and age

Feeding practices	Phone use ***N = 117*** (mean/SD)	No phone use ***N = 181*** (mean/SD)	B	95% CI	***P*** value
**Emotional regulation**	3.74 (1.70)	2.98 (1.73)	0.73	0.32; 1.14	< 0.001
**Balance and variety**	14.07 (1.90)	14.44 (1.80)	−0.37	− 0.80; 0.07	0.09
**Environment**	11.85 (3.15)	12.60 (3.10)	−0.77	−1.51; − 0.03	< 0.05
**Modeling**	12.54 (2.91)	13.56 (2.59)	−1.05	−1.69; − 0.41	< 0.001
**Monitoring**	14.83 (2.19)	14.39 (3.15)	0.49	−0.17; 1.15	0.14
**Pressure**	7.14 (3.20)	5.82 (3.58)	1.22	0.41; 2.03	< 0.01
**Restriction (Health)**	5.78 (3.22)	5.65 (3.21)	0.13	−0.64; 0.89	0.74
**Restriction (Weight)**	6.20 (3.88)	6.22 (4.34)	−0.06	−1.04; 0.90	0.90

Multiple regression analyses were used

*B* Beta, *CI* Confidence Interval

**Table 3:** Associations between phone use and shared family meals adjusted for parental educational status, ethnicity, and age

Shared family meals	Phone use (N)%	No phone use (N)%	OR	95% CI	***P*** value
**Breakfast**	58 (49.6)	121 (66.9)	0.50	0.31; 0.82	< 0.01
**Lunch**	34 (29.1)	72 (39.8)	0.63	0.38; 1.05	0.08
**Dinner**	67 (57.3)	129 (71.3)	0.57	0.35; 0.93	< 0.05

Logistic regression analyses were used

*OR* Odds Ratio, *CI* Confidence Interval

### Associations between phone use during mealtimes and feeding practice

Phone use during mealtimes was a significant predictor for the following feeding practices: emotional regulation (B = 0.73 (95% CI [0.32; 1.14])), family food environment (B = − 0.77 (95% CI [− 1.51; − 0.03])), modeling (B = − 1.05 (95% CI [− 1.69; − 0.41])) and pressure to eat (B = 1.22 (95% CI [0.41; 2.03])) (Table 2). After adjusting for parental educational status, ethnicity and age, these respective associations remained significant with a *p* value < 0.05.

### Associations between phone use and shared family meals

There were 50% lower odds of breakfast (95% CI [0.31; 0.82]) and 43% lower odds of dinner (95% CI [0.35; 0.93]) as daily shared family meals among the group using mobile phone compared to the group with no phone use during mealtimes (Table 3).

## Discussion

In this study, about 4 out of 10 parents reported various levels of phone use (meal distraction) during mealtimes. Parental phone use was associated with less use of positive parental feeding practices such as modelling and family food environment and more use of negative parental feeding practices like emotion regulation and use of pressure to eat. Descriptive analyses revealed more frequent family meals among the group with no phone use compared to the group with phone use during mealtimes. Dinner was the most commonly shared family meal for both groups. Finally, parental phone use during meals was associated with a lower frequency of daily family breakfast and dinner. Few studies have reported theses associations in this age group (toddlers) [15, 20].

When the child enters toddlerhood around 1 year of age, it becomes increasingly common to eat the same as the rest of the family and together with their family. This is a challenging time of transition. Parents increasingly engage in work-life again after parental leave, and consequently often have less time for other activities, making participation in family meals more challenging. We also know from a previous study that adults, including parents of toddlers, are increasingly exposed to distractions from their mobile phones during family mealtimes [14]. Since parental feeding practices and frequency of family meals are important when it comes to child eating development, the fact that parents’ attention is divided between the phone and the child, represents a growing dilemma. When parents use their mobile phone in the presence of their children, they are physically present, but distracted and probably unresponsive [29]. A small experimental study by Myruski et al. found that infants showed most distress when their mothers were disengaged and used their phones in the presence of their child [29]. We found that even in this higher educated sample, as much as 40% used their phone during meals to some extent. This is higher than reported by an earlier study that found that about 25% of mothers who participated had been distracted by technology use during mealtimes [30]. Radesky et al. [31] conducted an observational study in a fast-food restaurant assessing young children’s behavior in relation to the use of mobile devices by their parents. The parents’ use of mobile phones ranged from lying on the dining table to always being occupied with the phone. The study showed that 73% of the parents used their mobile device while eating, 29% used their mobile devices during the entire meal, and 15% looked at their smartphone mobile device while the child was still eating [31].

Parental meal distraction (e.g., mobile phone use) during meals may influence feeding practices, by less focus on the toddler’s food intake. It can further lead to negative modelling and use of food as a reward if the child gets impatient and for instance wants to leave the table [32]. An example may be if the parent is using food that the toddler prefers, but not necessarily healthy food, to keep the child quiet while they are using their phone. Also, monitoring, i.e., parents keeping track of child’s intake of less healthy food might be affected by phone use during meals since the attention is drawn away from the child. A previous study found that mobile device use among mothers was common and associated with fewer interactions with children during a structured interaction task, particularly nonverbal interactions, and that during introduction of an unfamiliar food they had less support and modelling from their mother [33]. It is fair to assume that the use of mobile phone during mealtimes may influence feeding practices. However, as these are cross-sectional associations, opposite directions of the associations should be considered, such as parental awareness of demonstrating healthy eating behavior (e.g., modelling) may also influence the use of mobile phone during mealtimes.

We found a higher frequency of all types of daily shared family meals among the group with no phone use compared to the group with phone use during mealtimes. Given that family meals have been reported to influence the eating habits of the child in a positive way [9], these findings are of interest. We found that parental meal distraction (phone use) was associated with lower frequency of daily family breakfast and dinner. It may be that if the parents are conscious about not using their phone during meals, this may be associated with other positive meal habits, such as the importance of having shared family meals. Another reason why the group with phone use during mealtimes has a lower frequency of shared family meals may be that families are busy and thus may be feeding their toddler earlier, and not actually eating together with their child. A consequence may be that they are multitasking, e.g. checking emails etc. while the child eats. A message that family meals are essential not only for dietary intake but also for socialization even before 1 year of age may be of importance for parents of young children.

Screen use in general (usually TV) during meals has been studied more than parental phone use during family meals, often linked to unfavorable outcomes such as childhood obesity [34, 35]. In a study by Jusiene et al., more than half of the children aged 2–5 were exposed to screens during meals: 34% several times per week or per month, and 22% daily or during every meal [34]. This study cannot be compared directly to our study, as 40% of the parents were distracted by their phone during meals with their toddlers, while if a TV is present during family meals, other unfavorable associations for the child may occur (e.g. increased daily screen time, increased intake of unhealthy foods) [34]. It is uncertain if phones uniquely influence food parenting practices and family meal frequency but given the habitual nature of phone use, e.g. that parents may not be aware of how often they are checking their phones during meals, it is possible that phone use may be less obvious than having a TV on during family meals. Raising awareness about the importance of putting phones away during mealtimes with young children is important. Future observational research exploring phone use during mealtimes would be an important next step investigate this.

### Strengths and limitations

The cross-sectional data analyzed, provide a snapshot of the study sample and cannot identify any causal associations and the findings may only be generalized to a population of parents with toddlers. The presence of fathers at mealtimes should not be neglected, as their presence are reported to be positively associated with less child distractions [14]. However, in this study we were not able to test statistically for potential differences between mothers and fathers due to the homogeneity of the sample (98.7% were mothers). Further, by dichotomizing the parents into those who use and do not use mobile phones during mealtimes, we have reduced the extent of variation in data and thus, may increase the risk of bias. Nevertheless, there are several strengths to be considered such as the relatively high sample size, the use of well-validated questionnaires and the fact that this study extends previous assumptions and research evidence.

## Conclusions

Mobile phone use is increasingly taking up time and attention and may thereby change the way we behave. This may also impact important parental tasks such as feeding practices and attention towards children during family meals. In the present study, 4 out of 10 parents with toddlers reported various levels of phone use during mealtimes. Parental phone use was associated with less use of positive parental feeding practices, more use of negative parental feeding practices and lower frequency of daily family breakfast and dinner. Our results highlight the importance of making parents aware of meal distractions like mobile phone use. Future studies should include fathers and explore observational and longitudinal data.

## Supplementary Information

**Additional file 1.** Food4toddlers questionnaire.

## Data Availability

Data will be made available on request from the corresponding author.

## Abbreviations

NCD’s: Non-communicable diseases
TV: Television
CFPQ: Comprehensive Feeding Practices Questionnaire
OR: Odds Ratio
CI: Confidence Interval
