# Supplementary material for: Parental phone use during mealtimes with toddlers and the associations with feeding practices and shared family meals: a cross-sectional study
Source: BMC Public Health. 2021 Apr 20;21:756. doi: 10.1186/s12889-021-10757-1 (PMC8056649; doi:10.1186/s12889-021-10757-1)
Supplement: Supplementary file 1 — Additional file 1. Food4toddlers questionnaire. [file 12889_2021_10757_MOESM1_ESM.pdf]

Thank you for participating in **Food4toddlers**!

The survey will take approximately 30 minutes to complete. If you do not have time to complete the whole survey at once, you can always stop and continue later. Some questions may seem alike but will provide us with a complete understanding at the end.

You may at any time point use the bottoms below to navigate back and forth in the survey.

Press next to continue.

Good luck!

All the best,
The Food4toddlers team,
University of Agder

Date when the survey was completed. Must be written as year-month-day. E.g. 2017-09-21

______

What is your relation to the child participating in the study?

(1) ☐ I am the mother

(2) ☐ I am the father

(3) ☐ I am neither. Specify ______

Are you living together with the father/mother of the child participating in the study?

(1) ☐ Yes

(2) ☐ No

The questions in this section applies to your child who is now approximately one year old. They involve background information, growth and development and the child's eating habits. Towards the end there will be some questions regarding parenting. You will need the child's health card to report the growth and height of the child.

Is your child twin/triplet?

(2) ☐ No

(1) ☐ Yes. Then you should fill in the form based on the oldest child.

What is the child's date of birth? Must be written in year-moth-day. E.g., 2016-09-30

______

What the baby's gender?

(1) ☐ Girl

(2) ☐ Boy

What was the child's weight and length at birth?

Weight (gram) _____

Length (cm) _____

Where is the child currently being taken care of during daytime?

(1) ☐ At home with the mother/father

(2) ☐ At home with childminder/trainee

(3) ☐ At childminder

(4) ☐ In family kindergarten

(6) ☐ In kindergarten

(5) ☐ Other, specify ______

How often does your child tend to eat the following meals during a week? (number of times/week)

	**Never/less frequent than every week**	**1/week**	**2/week**	**3/week**	**4/week**	**5/week**	**6/week**	**Every day**
Breakfast	(1) ☐	(2) ☐	(3) ☐	(4) ☐	(5) ☐	(6) ☐	(7) ☐	(8) ☐
Lunch	(1) ☐	(2) ☐	(3) ☐	(4) ☐	(5) ☐	(6) ☐	(7) ☐	(8) ☐
Snack before dinner	(1) ☐	(2) ☐	(3) ☐	(4) ☐	(5) ☐	(6) ☐	(7) ☐	(8) ☐
Dinner	(1) ☐	(2) ☐	(3) ☐	(4) ☐	(5) ☐	(6) ☐	(7) ☐	(8) ☐
Supper	(1) ☐	(2) ☐	(3) ☐	(4) ☐	(5) ☐	(6) ☐	(7) ☐	(8) ☐
Other meals/snacks	(1) ☐	(2) ☐	(3) ☐	(4) ☐	(5) ☐	(6) ☐	(7) ☐	(8) ☐

How often does your child have the following meals together with their family? (number of times/week)

	Never/less frequent than every week	1/week	2/week	3/week	4/week	5/week	6/week	Every day
Breakfast	(1) ☐	(2) ☐	(3) ☐	(4) ☐	(5) ☐	(6) ☐	(7) ☐	(8) ☐
Lunch	(1) ☐	(2) ☐	(3) ☐	(4) ☐	(5) ☐	(6) ☐	(7) ☐	(8) ☐
Snack before dinner	(1) ☐	(2) ☐	(3) ☐	(4) ☐	(5) ☐	(6) ☐	(7) ☐	(8) ☐
Dinner	(1) ☐	(2) ☐	(3) ☐	(4) ☐	(5) ☐	(6) ☐	(7) ☐	(8) ☐
Supper	(1) ☐	(2) ☐	(3) ☐	(4) ☐	(5) ☐	(6) ☐	(7) ☐	(8) ☐
Other meals/snacks	(1) ☐	(2) ☐	(3) ☐	(4) ☐	(5) ☐	(6) ☐	(7) ☐	(8) ☐

Does your child have any challenges relating to eating/food?

You can select more than one

(1) ☐ No, has no challenges

(2) ☐ Yes, bad appetite/nibbles

(3) ☐ Yes, likes only a few foods

(4) ☐ Yes, difficulties adjusting to the family diet

(5) ☐ Yes, allergic/intolerant to certain foods

(6) ☐ Yes, other poblems – describe _____

How often does the child currently get the following beverages?

	**Never/less frequent than every week**	**1-3 times/week**	**4-6 times/week**	**1time /day**	**2 times/day**	**3 times /day**	**4 times /day**	**5 times or more /day**
Breast milk	(1) ☐	(2) ☐	(3) ☐	(4) ☐	(5) ☐	(6) ☐	(7) ☐	(8) ☐

Breast-milk substitute	(1) ☐	(2) ☐	(3) ☐	(4) ☐	(5) ☐	(6) ☐	(7) ☐	(8) ☐
Regular sweet milk, all types (skim milk,semi-skim milk, whole milk)	(1) ☐	(2) ☐	(3) ☐	(4) ☐	(5) ☐	(6) ☐	(7) ☐	(8) ☐
Sour milk, all types (yogurt, biola, cultura etc.)	(1) ☐	(2) ☐	(3) ☐	(4) ☐	(5) ☐	(6) ☐	(7) ☐	(8) ☐
Chocolate milk, all types	(1) ☐	(2) ☐	(3) ☐	(4) ☐	(5) ☐	(6) ☐	(7) ☐	(8) ☐
Water	(1) ☐	(2) ☐	(3) ☐	(4) ☐	(5) ☐	(6) ☐	(7) ☐	(8) ☐

How often does the child currently get the following beverages?

	**Never/less frequent than every week**	**1-3/ week**	**4-6/ week**	**1/ day**	**2/ day**	**3/ day**	**4/ day**	**5 times or more/ day**
Squash, with sugar	(1) ☐	(2) ☐	(3) ☐	(4) ☐	(5) ☐	(6) ☐	(7) ☐	(8) ☐
Squash, artificially sweetened	(1) ☐	(2) ☐	(3) ☐	(4) ☐	(5) ☐	(6) ☐	(7) ☐	(8) ☐
Juice	(1) ☐	(2) ☐	(3) ☐	(4) ☐	(5) ☐	(6) ☐	(7) ☐	(8) ☐
Soda, with sugar	(1) ☐	(2) ☐	(3) ☐	(4) ☐	(5) ☐	(6) ☐	(7) ☐	(8) ☐
Soda, artificially sweetened	(1) ☐	(2) ☐	(3) ☐	(4) ☐	(5) ☐	(6) ☐	(7) ☐	(8) ☐

Smoothie, bought	(1) ▯	(2) ▯	(3) ▯	(4) ▯	(5) ▯	(6) ▯	(7) ▯	(8) ▯
Smoothie, homemade	(1) ▯	(2) ▯	(3) ▯	(4) ▯	(5) ▯	(6) ▯	(7) ▯	(8) ▯
Other	(1) ▯	(2) ▯	(3) ▯	(4) ▯	(5) ▯	(6) ▯	(7) ▯	(8) ▯

Does the child get homemade dinner or readymade (industrially produced) jarred/pouched baby food

(1) ▯ Only home made

(2) ▯ Mostly home made

(3) ▯ About half of each

(4) ▯ Most ready made

(5) ▯ Only redy made

How often does the child currently eat the following foods?

	**Never/less frequent than every week**	**1-3/ week**	**4-6/ week**	**1/ day**	**2/ day**	**3/day**	**4/ day**	**5 or more/ day**
Industrially produces porrage all types	(1) ▯	(2) ▯	(3) ▯	(4) ▯	(5) ▯	(6) ▯	(7) ▯	(8) ▯
Homemade porrage from whole meal flour or oats/oatmeal	(1) ▯	(2) ▯	(3) ▯	(4) ▯	(5) ▯	(6) ▯	(7) ▯	(8) ▯
Homemade porrage made from fine/white flour, rusk, semolina, rice or corn	(1) ▯	(2) ▯	(3) ▯	(4) ▯	(5) ▯	(6) ▯	(7) ▯	(8) ▯

How often does the child currently eat the following foods?

	**Never**	**Less than 1/ week**	**1-3/ week**	**4-6/week**	**1-2/ day**	**3 times or more/day**

Vegetables only	(1) ☐	(2) ☐	(3) ☐	(4) ☐	(5) ☐	(6) ☐
Industrially produced dinners jarred/puched containing vegetables and meat/chicken/turkey Industrifremstilt middag på	(1) ☐	(2) ☐	(3) ☐	(4) ☐	(5) ☐	(6) ☐
Industrially produced dinners jarred/puched containing vegetables and fish	(1) ☐	(2) ☐	(3) ☐	(4) ☐	(5) ☐	(6) ☐

How often does the child currently eat the following foods? Industrially produced

jarred/pouched dinners do not count here.

	**Never**	**Less than 1/week**	**1-3/ week**	**4-6 /week**	**1-2 /day**	**3 times or more/ day**
Meat, minced meat, meatballs, sausage etc.	(1) ☐	(2) ☐	(3) ☐	(4) ☐	(5) ☐	(6) ☐
Fish, fish pudding, fish ball/- cakes etc.	(1) ☐	(2) ☐	(3) ☐	(4) ☐	(5) ☐	(6) ☐
Pankakes	(1) ☐	(2) ☐	(3) ☐	(4) ☐	(5) ☐	(6) ☐
Pizza	(1) ☐	(2) ☐	(3) ☐	(4) ☐	(5) ☐	(6) ☐
Pasta	(1) ☐	(2) ☐	(3) ☐	(4) ☐	(5) ☐	(6) ☐
Rice	(1) ☐	(2) ☐	(3) ☐	(4) ☐	(5) ☐	(6) ☐
Bread	(1) ☐	(2) ☐	(3) ☐	(4) ☐	(5) ☐	(6) ☐

According to the bread scale below, how much wholemeal does the bread you usually serve your child contain?

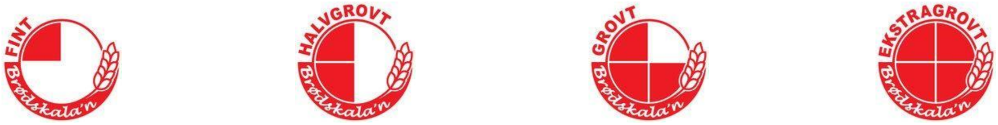

(1) ☐ (2) ☐ (3) ☐ (4) ☐

How many slices of bread does your child eat on a regular day? __

How often does your child currently eat the following foods? Industrially produced jarred/pouched dinners does not count here.

	Never	Less frequent than 1/week	1-3/ week	4-6/ week	1-2/ day	3 times or more / day
Potatoes	(1) ☐	(2) ☐	(3) ☐	(4) ☐	(5) ☐	(6) ☐
Boiled vegetables	(1) ☐	(2) ☐	(3) ☐	(4) ☐	(5) ☐	(6) ☐
Raw vegetables (including salad)	(1) ☐	(2) ☐	(3) ☐	(4) ☐	(5) ☐	(6) ☐
Fruit	(1) ☐	(2) ☐	(3) ☐	(4) ☐	(5) ☐	(6) ☐

How often does the child currently eat the following foods?

	Never	Less than 1/ week	1-3/ week	4-6/ week	1-2/ day	3 times or more/ day
Cakes, waffles, sweet biscuits	(1) ☐	(2) ☐	(3) ☐	(4) ☐	(5) ☐	(6) ☐
Dessert/ice cream	(1) ☐	(2) ☐	(3) ☐	(4) ☐	(5) ☐	(6) ☐

Chocolate	(1) ☐	(2) ☐	(3) ☐	(4) ☐	(5) ☐	(6) ☐
Bulk confectionery, gummy candy, other candy	(1) ☐	(2) ☐	(3) ☐	(4) ☐	(5) ☐	(6) ☐
Chips etc.	(1) ☐	(2) ☐	(3) ☐	(4) ☐	(5) ☐	(6) ☐

How often does the child currently eat the following? Include both raw, boiled and mashed vegetables (both homemade and industrially produced)

	**Never/less frequent than every week**	**1-3/ week**	**4-6/ week**	**1/ day**	**2/ day**	**3 times or more/ day**
Carrot	(1) ☐	(2) ☐	(3) ☐	(4) ☐	(5) ☐	(6) ☐
Rutabaga	(1) ☐	(2) ☐	(3) ☐	(4) ☐	(5) ☐	(6) ☐
Sweet potato	(1) ☐	(2) ☐	(3) ☐	(4) ☐	(5) ☐	(6) ☐
Cauliflower	(1) ☐	(2) ☐	(3) ☐	(4) ☐	(5) ☐	(6) ☐
Broccoli	(1) ☐	(2) ☐	(3) ☐	(4) ☐	(5) ☐	(6) ☐
Green salad	(1) ☐	(2) ☐	(3) ☐	(4) ☐	(5) ☐	(6) ☐
Spinach	(1) ☐	(2) ☐	(3) ☐	(4) ☐	(5) ☐	(6) ☐
Vegetable smoothie	(1) ☐	(2) ☐	(3) ☐	(4) ☐	(5) ☐	(6) ☐

How often does the child currently eat the following? Include both raw, boiled and mashed vegetables (both homemade and industrially produced)

	**Never/less frequent than every week**	**1-3/ week**	**4-6/ week**	**1/ day**	**2/ day**	**3 times or more/ day**
Cucumber	(1) ☐	(2) ☐	(3) ☐	(4) ☐	(5) ☐	(6) ☐
Tomato	(1) ☐	(2) ☐	(3) ☐	(4) ☐	(5) ☐	(6) ☐
Corn	(1) ☐	(2) ☐	(3) ☐	(4) ☐	(5) ☐	(6) ☐
Bell pepper	(1) ☐	(2) ☐	(3) ☐	(4) ☐	(5) ☐	(6) ☐
Peas/beans	(1) ☐	(2) ☐	(3) ☐	(4) ☐	(5) ☐	(6) ☐
Vegetable mix	(1) ☐	(2) ☐	(3) ☐	(4) ☐	(5) ☐	(6) ☐
Other	(1) ☐	(2) ☐	(3) ☐	(4) ☐	(5) ☐	(6) ☐

How often does the child currently eat the following fruits? (both homemade and industrially produced)

	**Never/less frequent than every week**	**1-3/ week**	**4-6/ week**	**1/ day**	**2/ day**	**3 times or more/ day**
Oranges/clementine etc.	(1) ☐	(2) ☐	(3) ☐	(4) ☐	(5) ☐	(6) ☐
Banana	(1) ☐	(2) ☐	(3) ☐	(4) ☐	(5) ☐	(6) ☐
Pear	(1) ☐	(2) ☐	(3) ☐	(4) ☐	(5) ☐	(6) ☐
Plum	(1) ☐	(2) ☐	(3) ☐	(4) ☐	(5) ☐	(6) ☐
Grapes	(1) ☐	(2) ☐	(3) ☐	(4) ☐	(5) ☐	(6) ☐

How often does the child currently eat the following fruits? (both homemade and industrially produced)

	**Never/less frequent than every week**	**1-3/ week**	**4-6/ week**	**1/ day**	**2/ day**	**3 times or more/ day**
**Kiwi**	**(1) ☐**	**(2) ☐**	**(3) ☐**	**(4) ☐**	**(5) ☐**	**(6) ☐**

**Melon**	**(1) ▯**	**(2) ▯**	**(3) ▯**	**(4) ▯**	**(5) ▯**	**(6) ▯**
**Mango**	**(1) ▯**	**(2) ▯**	**(3) ▯**	**(4) ▯**	**(5) ▯**	**(6) ▯**
**Berries, fresh or frozen**	**(1) ▯**	**(2) ▯**	**(3) ▯**	**(4) ▯**	**(5) ▯**	**(6) ▯**
**Other**	**(1) ▯**	**(2) ▯**	**(3) ▯**	**(4) ▯**	**(5) ▯**	**(6) ▯**

Does the child get any fish oil, vitamins, or other supplements?

(1) ▯ Yes

(2) ▯ No

Think of how it tends to be during a mealtime when answering. To what degree do you agree with the following statements:

	**Disagree**	**Slightly disagree**	**Neither agree or disagree**	**Slightly agree**	**Agree**
My child often tries new and different types of food	(1) ▯	(2) ▯	(3) ▯	(4) ▯	(5) ▯
My child does not trust new food	(1) ▯	(2) ▯	(3) ▯	(4) ▯	(5) ▯
If my child does not know what is in the food, he/she will not taste	(1) ▯	(2) ▯	(3) ▯	(4) ▯	(5) ▯
My child fears to eat things he/she has not eaten before	(1) ▯	(2) ▯	(3) ▯	(4) ▯	(5) ▯
My child is very picky on what foods he/she will eat	(1) ▯	(2) ▯	(3) ▯	(4) ▯	(5) ▯
My child eats almost all kinds of food	(1) ▯	(2) ▯	(3) ▯	(4) ▯	(5) ▯

The next questions regard food and food habits. Mark the answers you believe fit best. Some of the questions may not fit a one-year-old but try to answer to the best of the ability.

	Never	Seldom	Sometimes	Most times	Always
To what extent do you keep an eye on your child's intake of sweets (candy, ice cream, cakes, biscuits, etc.)?	(1) ☐	(2) ☐	(3) ☐	(4) ☐	(5) ☐
To what extent do you keep an eye on your child's intake of snacks (potato chips, Doritos, Cheetos etc.)?	(1) ☐	(2) ☐	(3) ☐	(4) ☐	(5) ☐
To what extent do you keep an eye on your child's intake of fatty foods your child eats?	(1) ☐	(2) ☐	(3) ☐	(4) ☐	(5) ☐
sugar sweetened beverages (soda, squash, iced tea etc.)?	(1) ☐	(2) ☐	(3) ☐	(4) ☐	(5) ☐
Do you allow your child to eat whatever he/she likes?	(1) ☐	(2) ☐	(3) ☐	(4) ☐	(5) ☐
Think of a dinner meal: do you let your child select the foods he/she likes among what is served for dinner?	(1) ☐	(2) ☐	(3) ☐	(4) ☐	(5) ☐

	Never	Seldom	Sometimes	Most often	Always
When you child is nagging, the first thing you do is give him/her something to eat or drink?	(5) ☐	(6) ☐	(7) ☐	(8) ☐	(9) ☐
Do you give you child something to eat or drink when he/she is bored, even if you do not believe he/she is hungry?	(5) ☐	(6) ☐	(7) ☐	(8) ☐	(9) ☐
When your child is angry or upset, do you give him/her something to drink even if you do not believe he/she is thirsty?	(5) ☐	(6) ☐	(7) ☐	(8) ☐	(9) ☐
If your child does not like what is served (e.g. for dinner), do you than prepare something different for him/her?	(5) ☐	(6) ☐	(7) ☐	(8) ☐	(9) ☐
Do you let you child eat snacks whenever he/she likes to?	(5) ☐	(6) ☐	(7) ☐	(8) ☐	(9) ☐
Is your child allowed to leave the table when he/she is full, even though the rest of the family has not finished eating?	(5) ☐	(6) ☐	(7) ☐	(8) ☐	(9) ☐
Do you encourage your child to eat healthy foods instead of unhealthy foods?	(5) ☐	(6) ☐	(7) ☐	(8) ☐	(9) ☐
	Disagree	Slightly disagree	Neither agree or disagree	Slightly agree	agree
Most of the foods I keep in the house is healthy	(5) ☐	(6) ☐	(7) ☐	(8) ☐	(9) ☐
I have a lot of snacks (potatochips, doritos ect.) in the house	(5) ☐	(6) ☐	(7) ☐	(8) ☐	(9) ☐

My child must always eat all the food on its plate	(5) ☐	(6) ☐	(7) ☐	(8) ☐	(9) ☐
I must assure my child does not eat too much fatty foods	(5) ☐	(6) ☐	(7) ☐	(8) ☐	(9) ☐
I offer my child his/her favorite food if he/she promises to behave nicely	(5) ☐	(6) ☐	(7) ☐	(8) ☐	(9) ☐
I let my child "help" prepare the food	(5) ☐	(6) ☐	(7) ☐	(8) ☐	(9) ☐
If I did not look after or set limitations to my son/daughter's food intake, he/she would eat too much if his/her favorite food	(5) ☐	(6) ☐	(7) ☐	(8) ☐	(9) ☐
Several healthy food items are available to my child at every meal that is served at home	(5) ☐	(6) ☐	(7) ☐	(8) ☐	(9) ☐
I offer my child sweets (candy, ice cream, cakes, biscuits etc.) as reward for good behavior	(5) ☐	(6) ☐	(7) ☐	(8) ☐	(9) ☐
I encourage my child to try new foods	(5) ☐	(6) ☐	(7) ☐	(8) ☐	(9) ☐
I talk to my child about why it is important to eat healthy foods	(5) ☐	(6) ☐	(7) ☐	(8) ☐	(9) ☐
I tell my child healthy food tastes good	(5) ☐	(6) ☐	(7) ☐	(8) ☐	(9) ☐
If I did not look after or set any limitations to my child's food intake, he/she would eat too much junk food (fast food, snacks, and sweets)	(5) ☐	(6) ☐	(7) ☐	(8) ☐	(9) ☐
I give my child small portions at meals so he/she will not get overweight of obese	(5) ☐	(6) ☐	(7) ☐	(8) ☐	(9) ☐

I my child tells me he/she is not hungry; I try to convince him/her to eat anyway	(5) ☐	(6) ☐	(7) ☐	(8) ☐	(9) ☐
I my child eat unusually much at a meal I try to limit his/her food intake at the next meal	(5) ☐	(6) ☐	(7) ☐	(8) ☐	(9) ☐
I limit my child's intake of foods than may cause him/her getting overweight or obese	(5) ☐	(6) ☐	(7) ☐	(8) ☐	(9) ☐
There are certain foods my child should not eat, as these may case him/her getting overweight or obese	(5) ☐	(6) ☐	(7) ☐	(8) ☐	(9) ☐
I hold back sweets/dessert as a reaction to bad behavior	(5) ☐	(6) ☐	(7) ☐	(8) ☐	(9) ☐
I have a lot of sweets (candy, ice cream, cakes, biscuits etc.) in the house	(5) ☐	(6) ☐	(7) ☐	(8) ☐	(9) ☐
I encourage my child to eat a varied diet (i.e. many different food items and dishes)	(5) ☐	(6) ☐	(7) ☐	(8) ☐	(9) ☐
If my child only east a small portion, I try to convince him/her to eat more	(5) ☐	(6) ☐	(7) ☐	(8) ☐	(9) ☐
I must assure my child does not eat too much of its favorite food	(5) ☐	(6) ☐	(7) ☐	(8) ☐	(9) ☐
I do not want my child to become overweight or obese, so I do not allow him/her to eat between meals	(5) ☐	(6) ☐	(7) ☐	(8) ☐	(9) ☐
I tell my child what to eat and what he/she shall not eat without explaining why	(5) ☐	(6) ☐	(7) ☐	(8) ☐	(9) ☐
I must be assured my child does not eat too many sweets (candy, ice cream, cakes, biscuits etc.)	(5) ☐	(6) ☐	(7) ☐	(8) ☐	(9) ☐
I am a role model to my child by eating healthy foods my self	(5) ☐	(6) ☐	(7) ☐	(8) ☐	(9) ☐

I try to eat healthy food when I am together with my son/daughter, even if this food is not my favorite food	(5) ☐	(6) ☐	(7) ☐	(8) ☐	(9) ☐
I try to show enthusiasm while I eat healthy food	(5) ☐	(6) ☐	(7) ☐	(8) ☐	(9) ☐
I show my child I really like eating healthy food	(5) ☐	(6) ☐	(7) ☐	(8) ☐	(9) ☐
When my child tells me he/she is finished eating I try to make him/her take a bite or two more	(5) ☐	(6) ☐	(7) ☐	(8) ☐	(9) ☐

How confident do you feel relating to the forthcoming:

	**Very confident**	**Slightly confident**	**Undecided**	**Slightly confident**	**Very confident**
That the food you provide your child is healthy	(1) ☐	(2) ☐	(3) ☐	(4) ☐	(5) ☐
That you can make your child eat enough	(1) ☐	(2) ☐	(3) ☐	(4) ☐	(5) ☐
That you can make you child taste different vegetables	(1) ☐	(2) ☐	(3) ☐	(4) ☐	(5) ☐
That you provide your child with the right amount of food	(1) ☐	(2) ☐	(3) ☐	(4) ☐	(5) ☐
That you can make your child try new foods	(1) ☐	(2) ☐	(3) ☐	(4) ☐	(5) ☐

To what extent do you agree with the following statements?

	**Disagree**	**Slightly disagree**	**Neither agree or disagree**	**Slightly agree**	**Agree**
It is calm when we eat dinner	(1) ☐	(2) ☐	(3) ☐	(4) ☐	(5) ☐
The dinners at our place is varied	(1) ☐	(2) ☐	(3) ☐	(4) ☐	(5) ☐
I offer the child food even though he/she did not like this food previously	(1) ☐	(2) ☐	(3) ☐	(4) ☐	(5) ☐

I often check my phone during meals	(1) ▯	(2) ▯	(3) ▯	(4) ▯	(5) ▯

How many hours a day does your child usually sit in front of the TV, computer/tablet, or smartphone?

(5) ▯ More than 4 hours

(1) ▯ 4 hours

(2) ▯ 3 hours

(3) ▯ 1-2 hours

(4) ▯ less than 1 hour

Part 2

Now follows some questions regarding you as a mother or father.

Which year are you born (for example 1989) ____

Which month are you born ____
(from 1-12):

What marital status do you currently have?

(1) ▯ Married

(2) ▯ Cohabitant

(3) ▯ Single

(4) ▯ Divorced/separated

(5) ▯ Widow/widower

(6) ▯ Other, describe _____

How many people are there in your household (usually)?

Number of adults __
Number of children __

What is the age of the children not participating in the study (who usually live at home). Answer in whole years and with comma; for example 3,5

______

What is currently your main activity?

(1) ▯ Work fulltime
(2) ▯ Work part time
(3) ▯ Stay at home
(4) ▯ Sick leave
(5) ▯ Leave
(6) ▯ Disability benefit
(7) ▯ Under occupational rehabilitation
(8) ▯ Student/school student
(9) ▯ Unemployed
(10) ▯ Other

What is your education? Select only one for the highest completed education.

(0) ▯ 9/10-year primary- or lower secondary school or less
(1) ▯ 9/10-year primary- or lower secondary school and folk high school or other one-year education
(2) ▯ Secondary education (high school/certificate of apprenticeship)
(3) ▯ Vocational education
(4) ▯ College or university education for 4 years or less
(5) ▯ College or university education for more than 4 years
(6) ▯ Other
(7) ▯ Do not know

What is the education of the child's other parent? Select only one for the highest completed education.

(0) ▯ 9/10-year primary- or lower secondary school or less
(1) ▯ 9/10-year primary- or lower secondary school and folk high school or other one-year education
(2) ▯ Secondary education (high school/certificate of apprenticeship)
(3) ▯ Vocational education
(4) ▯ College or university education for 4 years or less
(5) ▯ College or university education for more than 4 years

(6) ▯ Other

(7) ▯ Do not know

In which county do you live?

(1) ▯ Akershus

(2) ▯ Aust-Agder

(3) ▯ Buskerud

(4) ▯ Finnmark

(5) ▯ Hedmark

(6) ▯ Hordaland

(7) ▯ Møre og Romsdal

(8) ▯ Nord-Trøndelag

(9) ▯ Nordland

(10) ▯ Oppland

(11) ▯ Oslo

(12) ▯ Rogaland

(13) ▯ Sogn og Fjordane

(14) ▯ Sør-Trøndelag

(15) ▯ Telemark

(16) ▯ Troms

(17) ▯ Vest-Agder

(18) ▯ Vestfold

(19) ▯ Østfold

Have you experienced having problems managing running costs for food, transport, rent and such the last six months?

(1) ▯ No, never

(2) ▯ Yes, rarely

(3) ▯ Yes, occasionally

(4) ▯ Yes, often

How tall are you? Answer in cm

____

How much do you weigh? Answer in kg

___

Do you smoke?

(1) □ No, have never smoked on a regular basis

(2) □ No, have quit

(3) □ Yes, but not on a daily basis

(4) □ Yes, daily

During a normal week, how many days are you physically active for at least 30 minutes?

(1) □ None

(2) □ One day

(3) □ Two days

(4) □ Three days

(5) □ Four days

(6) □ Five days

(7) □ Six days

(8) □ Every day

On your spare time, how much time do you daily spend by the TV, computer/tablet or smartphone?

(6) □ more than 4 hours

(1) □ 4 hours

(2) □ 3 hours

(3) □ 1-2 hours

(4) □ Less than one hour

(5) □ Seldom/never

What is your ethnic background??

	**Yes**	**No**	**Do not know**

Were you born in Norway?	(1) ☐	(2) ☐	(3) ☐
Was your mother born in Norway?	(1) ☐	(2) ☐	(3) ☐
Was your father born in Norway?	(1) ☐	(2) ☐	(3) ☐
Was the child participating in the study born in Norway?	(1) ☐	(2) ☐	(3) ☐
Was the second parent of the child born in Norway?	(1) ☐	(2) ☐	(3) ☐

In the following there will be some questions concerning diet and food habits.

We ask how your eating habits usually is. Vi acknowledge that the diet varies from day today, therefore try to the best of the ability to provide an "average" of the way your eating habits has been the past year.

How often do you tend to eat the following meals during a week?

	**Never/less frequent than once/ week**	**1/week**	**2/week**	**3/week**	**4/ week**	**5/ week**	**6/ week**	**Every day**
Breakfast	(1) ☐	(2) ☐	(3) ☐	(4) ☐	(5) ☐	(6) ☐	(7) ☐	(8) ☐
Lunch	(1) ☐	(2) ☐	(3) ☐	(4) ☐	(5) ☐	(6) ☐	(7) ☐	(8) ☐
Snack before dinner	(1) ☐	(2) ☐	(3) ☐	(4) ☐	(5) ☐	(6) ☐	(7) ☐	(8) ☐
Dinner	(1) ☐	(2) ☐	(3) ☐	(4) ☐	(5) ☐	(6) ☐	(7) ☐	(8) ☐
Supper	(1) ☐	(2) ☐	(3) ☐	(4) ☐	(5) ☐	(6) ☐	(7) ☐	(8) ☐
Other meals/ snacks	(1) ☐	(2) ☐	(3) ☐	(4) ☐	(5) ☐	(6) ☐	(7) ☐	(8) ☐

How much do you drink of the following beverages?

	Never drink/rarely	1-3 glasses/ month	1-3 glasses/ month	4-6 glasses/ month	1-3 glasses/ day	4-6 glasses/ day	7 glasses/ day or more
Soda/squash with sugar	(1) ☐	(2) ☐	(3) ☐	(7) ☐	(8) ☐	(4) ☐	(9) ☐
Soda/squash, artificially sweetened	(1) ☐	(2) ☐	(3) ☐	(7) ☐	(8) ☐	(4) ☐	(9) ☐
Coffee	(1) ☐	(2) ☐	(3) ☐	(7) ☐	(8) ☐	(4) ☐	(9) ☐
Tea	(1) ☐	(2) ☐	(3) ☐	(7) ☐	(8) ☐	(4) ☐	(9) ☐
Alcohol	(1) ☐	(2) ☐	(3) ☐	(7) ☐	(8) ☐	(4) ☐	(9) ☐
Water	(1) ☐	(2) ☐	(3) ☐	(7) ☐	(8) ☐	(4) ☐	(9) ☐
Milk (all kinds)	(1) ☐	(2) ☐	(3) ☐	(7) ☐	(8) ☐	(4) ☐	(9) ☐

How many times do you eat the following foods?

	Never/rarer than every week	1-3 g/month	1-3 t/week	4-6 t/week	1 t/day	2 t/day	3 t/day	4 t/day or more
Fruit	(1) ☐	(9) ☐	(2) ☐	(3) ☐	(4) ☐	(5) ☐	(6) ☐	(8) ☐
Berries	(1) ☐	(9) ☐	(2) ☐	(3) ☐	(4) ☐	(5) ☐	(6) ☐	(8) ☐
Vegetables	(1) ☐	(9) ☐	(2) ☐	(3) ☐	(4) ☐	(5) ☐	(6) ☐	(8) ☐
Cakes, biscuits etc.	(1) ☐	(9) ☐	(2) ☐	(3) ☐	(4) ☐	(5) ☐	(6) ☐	(8) ☐
Desserts, ice cream etc.	(1) ☐	(9) ☐	(2) ☐	(3) ☐	(4) ☐	(5) ☐	(6) ☐	(8) ☐
Candy/chocolate/crisps etc.	(1) ☐	(9) ☐	(2) ☐	(3) ☐	(4) ☐	(5) ☐	(6) ☐	(8) ☐

How much do you agree to the following?

	Strongly disagree	Slightly disagree	Slightly agree	Strongly agree
I am constantly trying new and different foods	(1) ☐	(2) ☐	(3) ☐	(5) ☐
I do not trust unknown food	(1) ☐	(2) ☐	(3) ☐	(5) ☐
If I do not know what is in the food, I will not taste	(1) ☐	(2) ☐	(3) ☐	(5) ☐
I like food from different countries	(1) ☐	(2) ☐	(3) ☐	(5) ☐

How much do you agree to the following?

	Strongly disagree	Slightly disagree	Slightly agree	Strongly agree
In dinner parties I gladly try new foods	(1) ☐	(2) ☐	(3) ☐	(5) ☐

I am afraid for eat foods I have not eaten before	(1) ☐	(2) ☐	(3) ☐	(5) ☐
I am very picky to what kind of food I want to eat	(1) ☐	(2) ☐	(3) ☐	(5) ☐
I eat almost any kind of food	(1) ☐	(2) ☐	(3) ☐	(5) ☐

How often do you do the following?

	Never	Less than 1 t/week	1 t/week	2 t/week	3 t/week	4 t/week	5 t/week	6 t/week	7 t/week
Chop vegetables	(1) ☐	(2) ☐	(3) ☐	(4) ☐	(5) ☐	(6) ☐	(7) ☐	(8) ☐	(9) ☐
Chop fruit	(1) ☐	(2) ☐	(3) ☐	(4) ☐	(5) ☐	(6) ☐	(7) ☐	(8) ☐	(9) ☐
Prepare dinner from scratch	(1) ☐	(2) ☐	(3) ☐	(4) ☐	(5) ☐	(6) ☐	(7) ☐	(8) ☐	(9) ☐
Bake bread etc.	(1) ☐	(2) ☐	(3) ☐	(4) ☐	(5) ☐	(6) ☐	(7) ☐	(8) ☐	(9) ☐
Make home-made smoothie	(1) ☐	(2) ☐	(3) ☐	(4) ☐	(5) ☐	(6) ☐	(7) ☐	(8) ☐	(9) ☐
Find recipes online when I prepare food	(1) ☐	(2) ☐	(3) ☐	(4) ☐	(5) ☐	(6) ☐	(7) ☐	(8) ☐	(9) ☐
Use cooking videos etc. online when I prepare food	(1) ☐	(2) ☐	(3) ☐	(4) ☐	(5) ☐	(6) ☐	(7) ☐	(8) ☐	(9) ☐

Do you have the main responsibility for cooking at home?

(1) ☐ Yes

(2) ☐ No

(3) ☐ Shared responsibility

On a scale from 1 to 7, where 1 is not important and 7 is very important. Rank how important it is that the food I eat on a normal day:

	Not important						Very important
Is easy to prepare	(1) ☐	(2) ☐	(3) ☐	(4) ☐	(5) ☐	(6) ☐	(7) ☐
Tastes good	(1) ☐	(2) ☐	(3) ☐	(4) ☐	(5) ☐	(6) ☐	(7) ☐
Is not expensive	(1) ☐	(2) ☐	(3) ☐	(4) ☐	(5) ☐	(6) ☐	(7) ☐
Is familiar	(1) ☐	(2) ☐	(3) ☐	(4) ☐	(5) ☐	(6) ☐	(7) ☐
Contains a lot of fibre	(1) ☐	(2) ☐	(3) ☐	(4) ☐	(5) ☐	(6) ☐	(7) ☐
Is nutritious	(1) ☐	(2) ☐	(3) ☐	(4) ☐	(5) ☐	(6) ☐	(7) ☐

On a scale from 1 to 7, where 1 is not important and 7 is very important. Rank how important it is that the food I eat on a normal day:

	Not important						Very important
is easily accessible in the store	(1) ☐	(2) ☐	(3) ☐	(4) ☐	(5) ☐	(6) ☐	(7) ☐
Gives me a lot for my money	(1) ☐	(2) ☐	(3) ☐	(4) ☐	(5) ☐	(6) ☐	(7) ☐
smells good	(1) ☐	(2) ☐	(3) ☐	(4) ☐	(5) ☐	(6) ☐	(7) ☐
Can be prepared easily	(1) ☐	(2) ☐	(3) ☐	(4) ☐	(5) ☐	(6) ☐	(7) ☐
has a pleasant consistency	(1) ☐	(2) ☐	(3) ☐	(4) ☐	(5) ☐	(6) ☐	(7) ☐
is the same food that I ate when I was a child	(1) ☐	(2) ☐	(3) ☐	(4) ☐	(5) ☐	(6) ☐	(7) ☐

On a scale from 1 to 7, where 1 is not important and 7 is very important. Rank how important it is that the food I eat on a normal day:

	Not important						Very important
contains a lot of vitamins and minerals	(1) ☐	(2) ☐	(3) ☐	(4) ☐	(5) ☐	(6) ☐	(7) ☐
Look good	(1) ☐	(2) ☐	(3) ☐	(4) ☐	(5) ☐	(6) ☐	(7) ☐
Is rich in protein	(1) ☐	(2) ☐	(3) ☐	(4) ☐	(5) ☐	(6) ☐	(7) ☐
Takes minimal time to prepare	(1) ☐	(2) ☐	(3) ☐	(4) ☐	(5) ☐	(6) ☐	(7) ☐
Keeps me healthy	(1) ☐	(2) ☐	(3) ☐	(4) ☐	(5) ☐	(6) ☐	(7) ☐
is good for skin/teeth/hair/nails etc.	(1) ☐	(2) ☐	(3) ☐	(4) ☐	(5) ☐	(6) ☐	(7) ☐

On a scale from 1 to 7, where 1 is not important and 7 is very important. Rank how important it is that the food I eat on a normal day:

	Not important						Very important
Is what I usually eat	(1) ☐	(2) ☐	(3) ☐	(4) ☐	(5) ☐	(6) ☐	(7) ☐
Can be bought close to where I live or work	(1) ☐	(2) ☐	(3) ☐	(4) ☐	(5) ☐	(6) ☐	(7) ☐
Is cheap	(1) ☐	(2) ☐	(3) ☐	(4) ☐	(5) ☐	(6) ☐	(7) ☐

The following questions are about the store you usually do your grocery shopping.

Check the box that describes the general accessibility in the store:

	Not available	Slightly available	Moderately available	Very available
Berries	(1) ☐	(2) ☐	(3) ☐	(4) ☐
Fruit	(1) ☐	(2) ☐	(3) ☐	(4) ☐
Vegetables	(1) ☐	(2) ☐	(3) ☐	(4) ☐
Food pouches (baby food)	(1) ☐	(2) ☐	(3) ☐	(4) ☐

Children's biscuits, whole-grain	(1) ☐	(2) ☐	(3) ☐	(4) ☐
Children's biscuits, regular	(1) ☐	(2) ☐	(3) ☐	(4) ☐
Dinner jars for children	(1) ☐	(2) ☐	(3) ☐	(4) ☐
Candy/chocolate/crisps	(1) ☐	(2) ☐	(3) ☐	(4) ☐

How much do you agree to the following statements?

	**Disagree**	**Slightly Disagree**	**Undecided**	**Slightly Agree**	**Agree**
I usually take a shopping list with me	(1) ☐	(2) ☐	(3) ☐	(4) ☐	(5) ☐
I get affected by food advertisements	(1) ☐	(2) ☐	(3) ☐	(4) ☐	(5) ☐
I often buy baby food on jars and food pouches even though I have not planned it.	(1) ☐	(2) ☐	(3) ☐	(4) ☐	(5) ☐
I get affected by the packaging of the baby food	(1) ☐	(2) ☐	(3) ☐	(4) ☐	(5) ☐
In our household we use home delivered food crates for dinner	(1) ☐	(2) ☐	(3) ☐	(4) ☐	(5) ☐

How much do you agree to the following?

	**Disagree**	**Slightly Disagree**	**Undecided**	**Slightly Agree**	**Agree**
I often take with me foods that are highly visible in the store, even though I did not intend to buy it.	(1) ☐	(2) ☐	(3) ☐	(4) ☐	(5) ☐
I buy more if the store is clean and well organized.	(1) ☐	(2) ☐	(3) ☐	(4) ☐	(5) ☐
I often make use of 3 for 2 offers etc.	(1) ☐	(2) ☐	(3) ☐	(4) ☐	(5) ☐
If the store has placed foods in big stacks, I buy more of this item.	(1) ☐	(2) ☐	(3) ☐	(4) ☐	(5) ☐
I am consciously searching for offers where I got a lot for my money	(1) ☐	(2) ☐	(3) ☐	(4) ☐	(5) ☐
I buy what I need and do not get affected buy where the item is placed in the store.	(1) ☐	(2) ☐	(3) ☐	(4) ☐	(5) ☐

The following questions are about the food availability at home and about meal planning.

How much of the following foods do you usually have available at home (both fresh and frozen). Check the box that describes the general access to food in your home:

	**None**	**Little**	**Some**	**A lot**	**Very much**
Berries	(1) ☐	(2) ☐	(5) ☐	(3) ☐	(4) ☐
Fruit	(1) ☐	(2) ☐	(5) ☐	(3) ☐	(4) ☐
Vegetables	(1) ☐	(2) ☐	(5) ☐	(3) ☐	(4) ☐
Candy/chocolate/crisps etc.	(1) ☐	(2) ☐	(5) ☐	(3) ☐	(4) ☐
Soda	(1) ☐	(2) ☐	(5) ☐	(3) ☐	(4) ☐
Fish/shellfish	(1) ☐	(2) ☐	(5) ☐	(3) ☐	(4) ☐
Chicken	(1) ☐	(2) ☐	(5) ☐	(3) ☐	(4) ☐
Meat	(1) ☐	(2) ☐	(5) ☐	(3) ☐	(4) ☐

	**Easily accessible on the kitchen counter etc.**	**Accessible in the cupboard/fridge/freezer etc.**	**Limited accessibility far into the cupboard/basement/freezer etc.**
Berries	(1) ☐	(2) ☐	(3) ☐
Fruit	(1) ☐	(2) ☐	(3) ☐
Vegetables	(1) ☐	(2) ☐	(3) ☐
Candy/chocolate/crisps etc.	(1) ☐	(2) ☐	(3) ☐
Soda	(1) ☐	(2) ☐	(3) ☐
Cakes/biscuits etc.	(1) ☐	(2) ☐	(3) ☐

During the last month, have you done any of the following with your family?

	Never	Rarely	Sometimes	Frequently	Very frequently
Ate at a family restaurant (e.g., Pizza Hut or Applebee's)	(2) ☐	(3) ☐	(4) ☐	(5) ☐	(6) ☐
Ate at a fast-food restaurant (e.g., McDonalds or Burger King)	(2) ☐	(3) ☐	(4) ☐	(5) ☐	(6) ☐
Had take-away food at home (e.g., Domino's or sushi)	(2) ☐	(3) ☐	(4) ☐	(5) ☐	(6) ☐
Had a ready-meal at home (e.g., frozen pizza etc.)	(2) ☐	(3) ☐	(4) ☐	(5) ☐	(6) ☐

To what degree are these statements true?

	Absolutely not	Partly true	Quite true	True	Very true
I plan the menu ahead of the coming week	(1) ☐	(2) ☐	(3) ☐	(4) ☐	(5) ☐
I make shopping lists for one week at the time	(1) ☐	(2) ☐	(3) ☐	(4) ☐	(5) ☐
I make healthy meals even if I have few available ingredients	(1) ☐	(2) ☐	(3) ☐	(4) ☐	(5) ☐
I prepare meals in advance (during the weekend or spare time)	(1) ☐	(2) ☐	(3) ☐	(4) ☐	(5) ☐
I prepared double portions of the food to save for later (frozen or stored in the fridge)	(1) ☐	(2) ☐	(3) ☐	(4) ☐	(5) ☐

Where have you received information about diet and nutrition to the child from 6 months of age and until now, and how do you evaluate this information?

	Not received any information	Very useful	Useful	Not very useful	Not useful
Health Centre	(1) ▯	(2) ▯	(3) ▯	(4) ▯	(5) ▯
Health professionals outside of the Health Centre	(1) ▯	(2) ▯	(3) ▯	(4) ▯	(5) ▯
Family/acquaintances	(1) ▯	(2) ▯	(3) ▯	(4) ▯	(5) ▯
Public web sites (e.g. the Norwegian Directorate of Health or "Matportalen")	(1) ▯	(2) ▯	(3) ▯	(4) ▯	(5) ▯
Blogs	(1) ▯	(2) ▯	(3) ▯	(4) ▯	(5) ▯
Other web sites	(1) ▯	(2) ▯	(3) ▯	(4) ▯	(5) ▯

Finally, based on your child's Health Card, fill in the child's weight and length

at approximately 6-7 months of age

The date the measurements were completed (For example 2017-08-31) _____

Weight (grams) _____

Length (cm) _____

at approximately 9-10 months of age

The date the measurements were completed (For example 2017-08-31) _____

Weight (grams) _____

Length (cm) _____

at approximately on one year of age

The date the measurements were completed (For example 2017-08-31) _____

Weight (grams) _____

Length (cm) _____
